# A robust, single-injection method for targeted, broad-spectrum plasma metabolomics

**DOI:** 10.1007/s11306-017-1264-1

**Published:** 2017-09-04

**Authors:** Kefeng Li, Jane C. Naviaux, A. Taylor Bright, Lin Wang, Robert K. Naviaux

**Affiliations:** 1 The Mitochondrial and Metabolic Disease Center, University of California, San Diego, School of Medicine, 214 Dickinson St., Bldg CTF, Rm C102, San Diego, CA 92103-8467 USA; 2 Department of Medicine, University of California, San Diego, School of Medicine, 214 Dickinson St., Bldg CTF, Rm C102, San Diego, CA 92103-8467 USA; 3 Department of Pediatrics, University of California, San Diego, School of Medicine, 214 Dickinson St., Bldg CTF, Rm C102, San Diego, CA 92103-8467 USA; 4 Department of Pathology, University of California, San Diego, School of Medicine, 214 Dickinson St., Bldg CTF, Rm C102, San Diego, CA 92103-8467 USA; 5 Department of Neurosciences, University of California, San Diego, School of Medicine, 214 Dickinson St., Bldg CTF, Rm C102, San Diego, CA 92103-8467 USA

**Keywords:** Targeted metabolomics, LC-MS/MS, Broad-spectrum, Hydrophilic interaction chromatography, Chronic fatigue syndrome, Validation

## Abstract

**Background:**

Metabolomics is a powerful emerging technology for studying the systems biology and chemistry of health and disease. Current targeted methods are often limited by the number of analytes that can be measured, and/or require multiple injections.

**Methods:**

We developed a single-injection, targeted broad-spectrum plasma metabolomic method on a SCIEX Qtrap 5500 LC-ESI-MS/MS platform. Analytical validation was conducted for the reproducibility, linearity, carryover and blood collection tube effects. The method was also clinically validated for its potential utility in the diagnosis of chronic fatigue syndrome (CFS) using a cohort of 22 males CFS and 18 age- and sex-matched controls.

**Results:**

Optimization of LC conditions and MS/MS parameters enabled the measurement of 610 key metabolites from 63 biochemical pathways and 95 stable isotope standards in a 45-minute HILIC method using a single injection without sacrificing sensitivity. The total imprecision (CV_total_) of peak area was 12% for both the control and CFS pools. The 8 metabolites selected in our previous study (PMID: 27573827) performed well in a clinical validation analysis even when the case and control samples were analyzed 1.5 years later on a different instrument by a different investigator, yielding a diagnostic accuracy of 95% (95% CI 85–100%) measured by the area under the ROC curve.

**Conclusions:**

A reliable and reproducible, broad-spectrum, targeted metabolomic method was developed, capable of measuring over 600 metabolites in plasma in a single injection. The method might be a useful tool in helping the diagnosis of CFS or other complex diseases.

**Electronic supplementary material:**

The online version of this article (doi:10.1007/s11306-017-1264-1) contains supplementary material, which is available to authorized users.

## Introduction

Metabolomics has recently emerged as a useful tool with broad applications in clinical and translational research such as disease biomarker discovery, clinical drug safety evaluation and precision medicine (Patti et al. 2012; Becker et al. 2012). It can be performed using either untargeted or targeted approaches. Untargeted metabolomics generally requires high-resolution time-of-flight (TOF) instruments to generate precision accurate mass data for all the detectable ions (Spalding et al. 2016). Accurate metabolite identification in untargeted metabolomics is time-consuming and hindered by the requirement for extensive accurate mass databases and sophisticated MS deconvolution and metabolite identification steps. Even though thousands of features (molecular ions) are captured in untargeted metabolomics, only a limited number of metabolites, typically fewer than 0.2–5% of the features, can actually be identified and confirmed (Schymanski and Neumann 2013).

Targeted metabolomics offers several advantages over the untargeted approach. Generally, the targeted metabolomics is conducted using a triple quadrupole mass spectrometer (QqQ, also designated as MS/MS) in multiple reaction monitoring (MRM) mode, which is usually less expensive and more robust than TOF instruments (Ferrario et al. 2016). Since the MRM transitions are specifically optimized to the targeted metabolites using the authentic standards, data analysis is easier. Moreover, MRM-based targeted metabolomics is intrinsically more sensitive, selective, and quantitative, and has a broader dynamic range of 5–6 orders of magnitude than MS based on untargeted metabolomics in complex biological samples using TOF or QTOF instruments (Morin et al. 2013; Dillen et al. 2012).

MRM based targeted analysis by tandem mass spectrometry has become a routine diagnostic tool in clinical laboratories such as vitamin D analysis and the screening of inborn errors of metabolism for newborns (Farrell et al. 2012; la Marca 2014). However, the small number of metabolites measured by typical targeted methods (up to 300 metabolites for single-injection methods) limits the use of targeted metabolomics as a discovery toolbox (Yuan et al. 2012; Bajad et al. 2006). A single LC-MS/MS platform targeting a large scale of metabolites is highly desirable to fulfill different application requirements, reduce the cost and increase the productivity of metabolomics.

The analysis of complex samples requires efficient chromatographic separation of the metabolites prior to MS to reduce ion suppression and improve signal sensitivity. Hydrophilic interaction chromatography (HILIC) has proved to be an effective method for the separation of water-soluble metabolites that are only minimally retained in the reversed-phase liquid chromatography (RPLC) (Bajad et al. 2006; Ivanisevic et al. 2013). HILIC has also been optimized for the separation of phospholipids (Losito et al. 2013; Schwalbe-Herrmann et al. 2010).

Chronic fatigue syndrome (CFS) is a complex, multisystem disease that affects more than 2 million people in the United States (Prins et al. 2006). The symptoms of CFS include, but not limited to: post-exertional malaise, disabling fatigue, chronic pain, and nonrestorative sleep (Clayton 2015). No biochemical diagnostic lab test is currently available. Our previous study identified unique metabolic signatures in the plasma of patients with CFS using the targeted metabolomic approach (Naviaux et al. 2016). In this study, we described the full method with details on key analytical procedures such as method optimization, MRM transitions, linearity and carryover which would help other researchers in the field to adapt and apply this method to their own work. To confirm the inter-instrument and inter-tester reproducibility of our metabolomic method, we also reanalyzed 40 plasma samples from the previous CFS study on a different instrument by a different investigator.

## Materials and methods

### Chemicals and material

Unlabeled chemical standards were purchased from Sigma–Aldrich, Cayman Chemical, TCI America, Fisher Scientific, or Avanti Polar Lipids. HPLC grade (NH_4_)_2_CO_3_ (Catalog No. A651-500) and ACS grade NH_4_OH (Catalog No. AC423300250) were from ACROS Organics, Fisher Scientific. LC-MS grade water (Catalog No. LC365) and acetonitrile (Catalog No. LC015) were purchased from Honeywell Burdick & Jackson. Commercial stable isotopes were obtained from Cambridge Isotope. Blood collection tubes including Lithium heparin (Catalog No. 367,884), sodium heparin (Catalog No. 367,871), dipotassium (K_2_)-EDTA (Catalog No. 367,861) and serum separator tube (SSTs, Order No. 367,983) were from BD. Custom-synthesized uniformly ^13^C-labeled metabolite internal standards were produced via metabolic labeling by growing *Escherichia coli* NCM 3277, *Caenorhabditis elegans* N2, and *Komagataella phaffii* with ^13^C_6_-glucose and ^13^C-bicarbonate as sole carbon sources.

### Metabolites extraction

All human samples were collected with signed informed consent in accordance with a human subject protocol approved by University of California, San Diego Institutional Review Board (IRB #14-0072). Plasma samples (90 µl) were mixed with 5 µl of commercial internal standards and 5 µl of custom-synthesized ^13^C labeled standards. The mixture was then extracted with 400 µl of cold (−20 °C) methanol: acetonitrile (50:50 MEOH:ACN). The extract was stored at −80 °C prior to LC-MS/MS. Detailed procedures were described in the online Supplemental Materials.

### LC-MS/MS analysis

Details for the optimization of LC and MS/MS conditions were provided in the online Supplemental Materials. The optimal conditions are described below.

LC-MS/MS analysis was performed using a UFLC XR HPLC system (LC-20AD, Shimadzu) coupled to a Turbo V electrospray ionization source and a Qtrap 5500 mass spectrometer (SCIEX). The samples were separated in HILIC mode using a polymer-based NH_2_ 4 µm, 250 × 2 mm column (Asahipak NH_2_P-40 2E, Shodex). The optimal LC conditions were as follows: Mobile phase A: 95% H_2_O with 20 mM (NH_4_)_2_CO_3_ and 5% ACN, pH 9.8. Mobile phase B: 100% ACN. The gradient was: 0–3.5 min 95% B, 3.6–8 min 85% B, 8.1–13 min 75% B, 14–30 min 0% B, 31–41 min 95% B, 41.1 min stop. The flow rate was 200 µl/min and the injection volume was 10 µl. Typical pump pressures ranged from 850 to 2800 psi over the course of the run.

The MS/MS detection was performed using electrospray ionization (ESI) and by scheduled multiple reaction monitoring (MRM). All 610 metabolites and 95 internal standards were targeted in a single injection using both negative and positive modes with rapid polarity switching (50 ms) and advanced scheduled MRM algorithm. The ESI source conditions were set as follows: electrospray voltage of −4500 V for negative mode and 5500 V for positive mode, source temperature of 500 °C, curtain gas of 30, ion source gas 1 and gas 2 of 35 psi, respectively. The mass transitions and compound-dependent parameters were optimized using the authentic standards and listed in Table S1. This table is formatted for easy compatibility with Analyst 1.6 software (SCIEX).

### Analytical validation

A balanced precision experiment was performed to evaluate the reproducibility of the method using five replicates of the control and pooled CFS plasma analyzed on each of 5 days (Grant and Hoofnagle 2014). The mean intra-assay (CV_intra_), inter assay (CV_inter_) and total CVs for each pool were calculated. The linearity was evaluated using 5-point admixtures of control and CFS pools (100, 75, 50, 25, and 0% of the CFS pool in the mixture). Duplicates were performed for each point. The peak quality and carryover were estimated using the pooled adult human plasma spiked with stable isotope internal standards (Quality control samples, QC). Effect of blood collection tubes was evaluated using the samples collected by four types of tubes including Li-heparin, Na-heparin, K2-EDTA and serum separator tube (SSTs).

Details for the procedures of analytical validation were described in online Supplemental Materials.

### Inter-instrument and inter-tester reproducibility

Clinical validation consisted of a cohort of 22 male CFS (53 ± 2.8 years old, mean ± SEM, range 21–67 years) and 18 male controls (53 ± 3.5 years old, mean ± SEM, range 23–69 years), who were enrolled in our previous study (Naviaux et al. 2016). These plasma samples were stored in −80 °C for about 1.5 years and reanalyzed on a different LC-MS/MS system by a different investigator. Metabolomic data was log 2 transformed and analyzed by partial least squares discriminant analysis (PLS-DA) in MetaboAnalyst (http://www.metaboanalyst.ca). Eight metabolites were selected for testing as a diagnostic classifier in the original study (Naviaux et al. 2016). Overfitting was minimized by selecting classifier metabolites to interrogate altered pathways using a combination of random forest analysis (Breiman 2001), forward selection, and backward elimination methods to identify a set of metabolites-a “biosignature”-that performed well as a diagnostic classifier by receiver operator characteristic (ROC) curve analysis (Szymanska et al. 2012; Xia et al. 2013). These same eight metabolites were then tested in the current study, after repeating the sample metabolomic analysis in this study, 1.5 years later. Classifier robustness was estimated by repeated double cross-validation (rdCV) and permutation testing 1000 times in MetaboAnalyst (Table S2). The correlation of peak area, z-scores and pathway impact scores between two analyses were also used to check the reproducibility of the method for the diagnosis of CFS.

### Data analysis

The peak quality evaluation metrics including retention time (RT), peak area, peak width, peak width at 50% height, and signal to noise ratio (S/N) were generated in MultiQuant 3.0 (SCIEX) and log 2 transformed prior to statistical analysis. Coefficient of variation (CV), Pearson correlation, frequency distribution and linear regression analysis were conducted in Graphpad Prism 6.0. PLS-DA, heatmap, volcano plot analysis and ROC curve analysis were performed in metaboanalyst (http://www.metaboanalyst.ca).

## Results

### Targeted metabolites

**Fig. 1:**
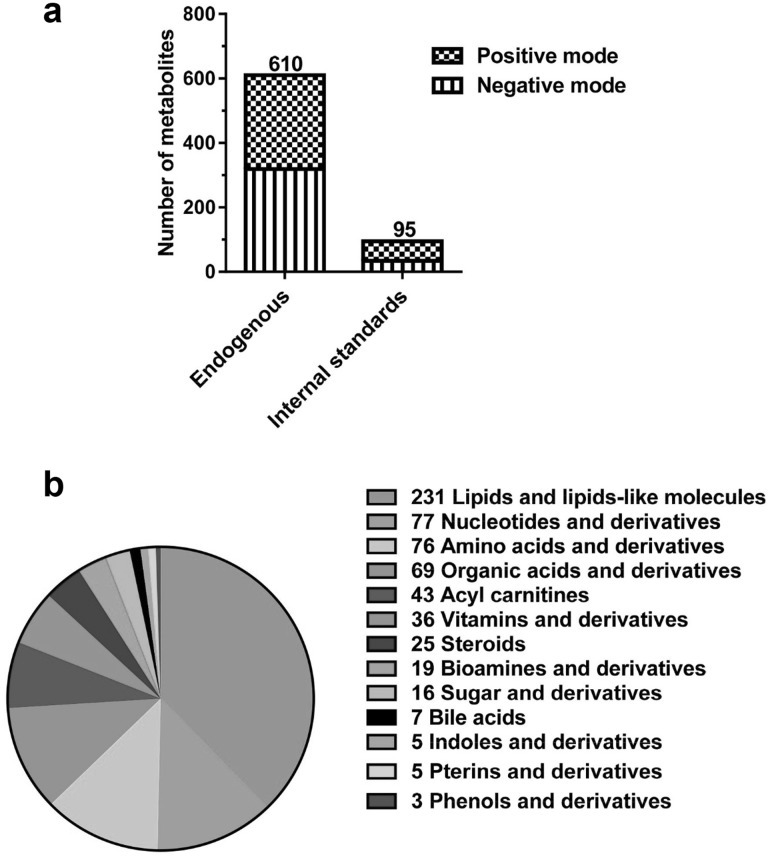
Total 705 metabolites were targeted on both negative and positive mode in a single run. **a** The number of targeted metabolites in the method. **b** Targeted endogenous metabolites with diverse chemical classes

Purified authentic standards were used to determine the MRM transitions, compound-specific parameters, and column retention times. A total of 610 endogenous metabolites were finally selected based on the availability of standards and reliable quantification on the LC columns (Fig. 1a, b). These metabolites cover a broad range of chemical classes and known to be essential in a variety of health and disease states. Table S1 lists the optimal MRM transition, source parameters, chemical class and the metabolic pathway of each metabolite.

The application of stable isotopes in metabolomics helps the correction of extraction loss, matrix effect, ion suppression and MS signal drift. They are also critical for inter-batch normalization for large-scale metabolomic studies. In this study, a mix of commercial stable isotopes and in-house prepared fully ^13^C-labeled metabolites were created and 95 stable isotope standards were targeted (Fig. 1a). In total, 705 metabolites were analyzed using the single injection method.

### Method optimization

A detailed description of optimization steps and analysis is described in the Online Supplemental Results.

#### Columns

Aminopropyl columns from three different manufacturers: Luna NH_2_ (Phenomenex), inertsil NH_2_ (GL Science) and Asahipak NH_2_P-40 (Shodex) were evaluated. Based on our observation, we found the polymer-based column from Shodex to have a longer lifetime, typically over 1000 injections, and to be more stable at high pH than the silica based aminopropyl columns. Further optimization was conducted using a Shodex 250 × 2 mm aminopropyl column.

#### LC conditions

**Fig. 2:**
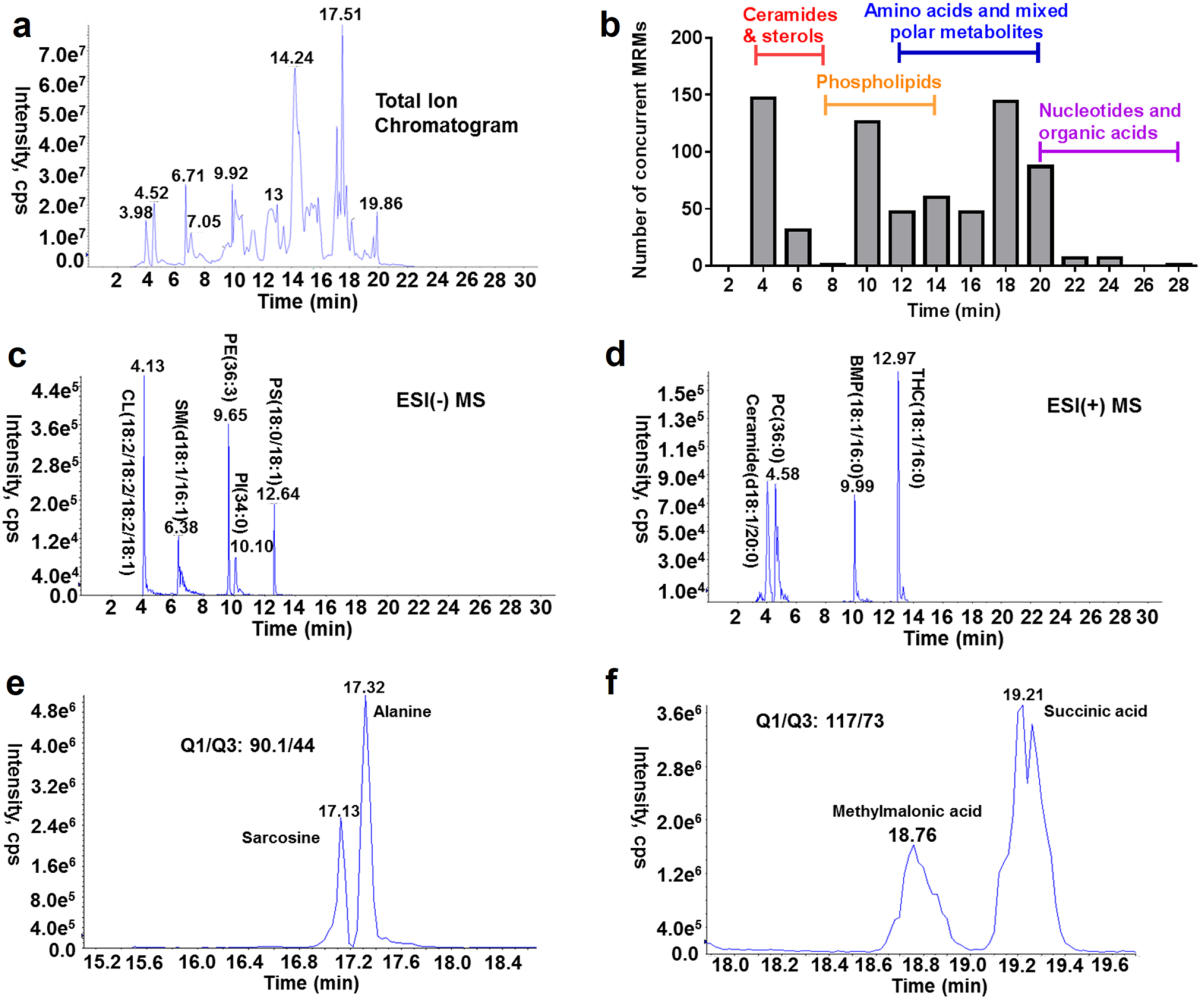
The chromatographic separation of 705 targeted metabolites on a shodex polymer-based aminopropyl column in HILIC mode in a single run. **a** The total ion chromatogram (TIC) of a quality control plasma extract analyzed using the developed method; **b** the frequency histogram of retention time (RT). The first metabolite was eluted at about 4 min. The bin size was set to 2-min; **c, d** the separation of different classes of lipids; **e** the baseline separation of alanine and its isomer sarcosine; **f** the separation of methylmalonic acid (MMA) and succinic acid. *CL* cardiolipin, *SM* sphingomyelin, *PE* phosphatidylethanolamine, *PI* phosphatidylinositol, *PC* phosphatidylcholine, *PS* phosphatidylserine, *BMP* bis(monoacylglycero)phosphate, *THC* trihexosylceramide, *HILIC* hydrophilic interaction liquid chromatography

Mobile phase compositions, pH, and oven temperatures were carefully investigated to achieve the best separation (Fig. S1 and S2). As shown in Fig. 2a, the targeted 705 analytes were eluted across the entire gradient. In a given 2-min window, the maximum number of compounds scanned was only about 150 (Fig. 2b). Some representative examples are shown in Fig. 2c–f. Six different classes of membrane lipids were separated on the HILIC column including ceramides, glycosphingolipids, sphingomyelins, cardiolipins, phospholipids and bis(monoacylglycero)phosphates (Fig. 2c, d). The method also showed excellent separation of four types of glycerophospholipids including phosphatidylcholine (PC, RT: 4.58 min), phosphatidylethanolamine (PE, RT: 9.65 min), phosphatidylinositol A (PI, RT: 10.10 min), and phosphatidylserine (PS, RT: 12.64) (Fig. 2c, d). Baseline separation of several isobaric metabolites, including alanine and sarcosine (Fig. 2e) and methylmalonic acid and succinic acid (Fig. 2f) was achieved using this method.

#### MS/MS conditions

We optimized the source temperature and ion source gas using quality control (QC) plasma extract lots to determine the conditions suitable for our diverse classes of targeted metabolites. Optimal source parameters for our method were ion source gas at 35 psi, and source temperature set at 500 °C (Fig. S3). Traditional LC-MS/MS based metabolomics often requires two separate injections for negative and positive ions (Pluskal et al. 2010). In combination with the rapid polarity switching and scheduled MRM algorithm, we quantified all 705 MRM transitions in a single run without significantly reducing the sensitivity (Fig. S4).

### Analytical validation

#### Peak quality evaluation

**Fig. 3:**
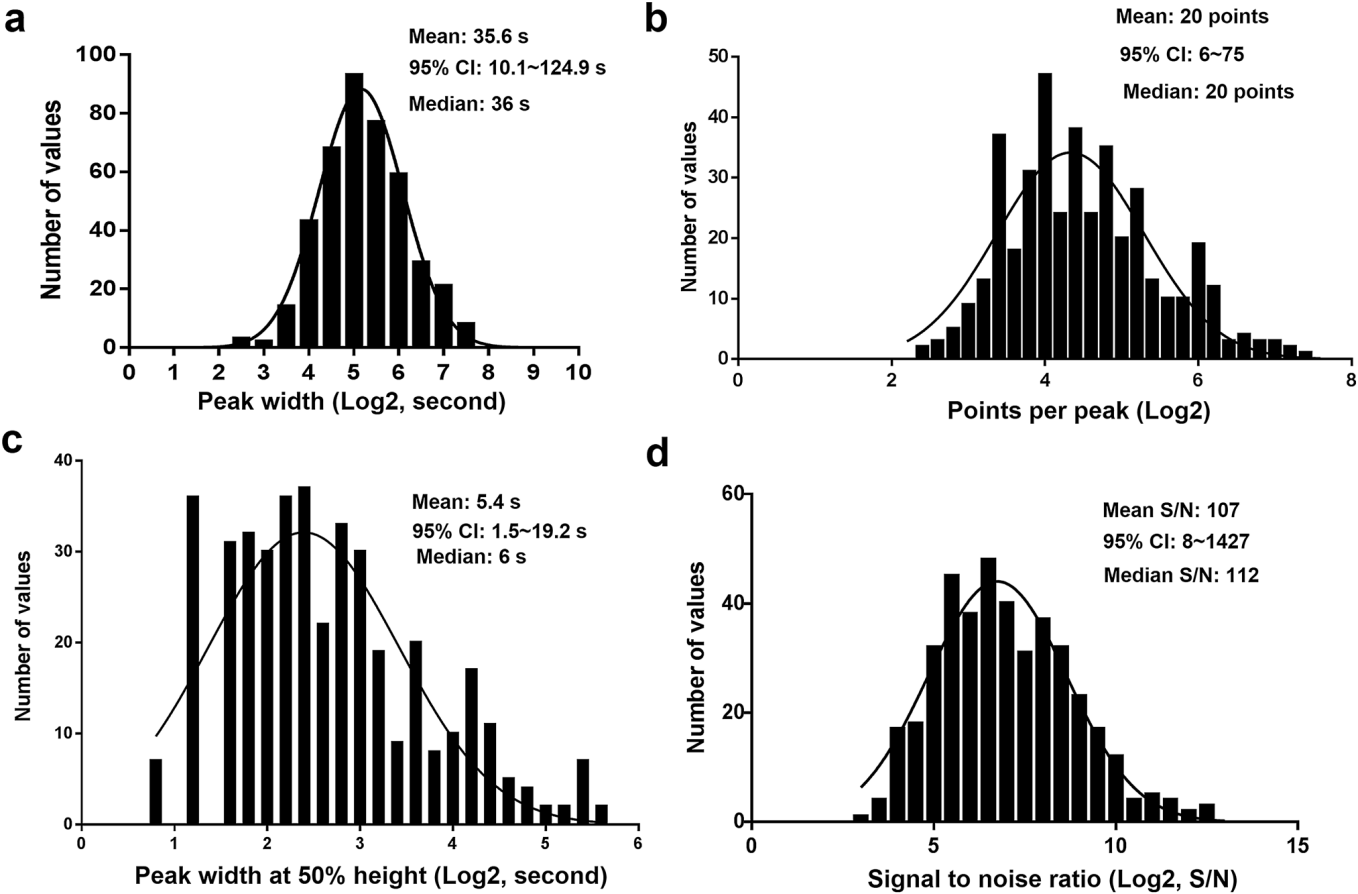
Frequency distribution analysis of peak quality for the targeted metabolites in human plasma. **a** Histogram of peak width, **b** histogram of points across the peaks, **c** histogram of peak width at 50% of peak height and **d** histogram of signal to noise ratio (S/N). Pooled plasma samples were analyzed using the developed metabolomic method. The peak quality was assessed using Multiquant 3.0. Histograms and best-fit Gaussian distributions were plotted in Graphpad Prism 6.0 in log space and the mean and 95% confidence interval (CI) were provided in the linear space

To characterize the quality of the peaks generated using the developed method, we performed the frequency distribution analysis of peak area, width, width at 50% of the peak height, points across the peak, and signal to noise ratio (S/N) for all metabolites detected in plasma. All the peak metrics showed a good fit to a lognormal distribution (Fig. 3). Peak width is an important factor that determines the actual dwell time spent on the peak and points across the peak in scheduled MRM algorithm. The average peak width of the targeted analytes was 35.6 s with 95% confidence interval (CI) between 10.1 and 124.9 s and the mean of peak width at 50% of peak height was 5.4 s (Fig. 3a, c). The average number of data points across the peak was 20, which ensures the accurate and reproducible quantification of the analytes (Fig. 3b). The 95% CI of signal to noise (S/N) for the targeted metabolites was between 8 and 1427 with the mean of 107 (Fig. 3d).

#### Reproducibility

Generating reproducible results is essential for the success of large-scale metabolomic studies that usually involve hundreds of samples and require multiple batches to complete the analysis of a large cohort of samples. The average CV for intra-batch chromatographic retention time (RT) variation was 0.11% (95% CI 0.02–0.34%) for the control pool and 0.12% (95% CI 0.02–0.39) for the CFS pool. The inter-batch RT variation was also very small, with average CVs of 0.14% (95% CI 0.02–0.44%) for the control pool and 0.15% (95% CI 0.02–0.54%) for the CFS pool. This is particularly important because the scheduled MRM algorithm relies on the stability of analyte retention time.

**Fig. 4:**
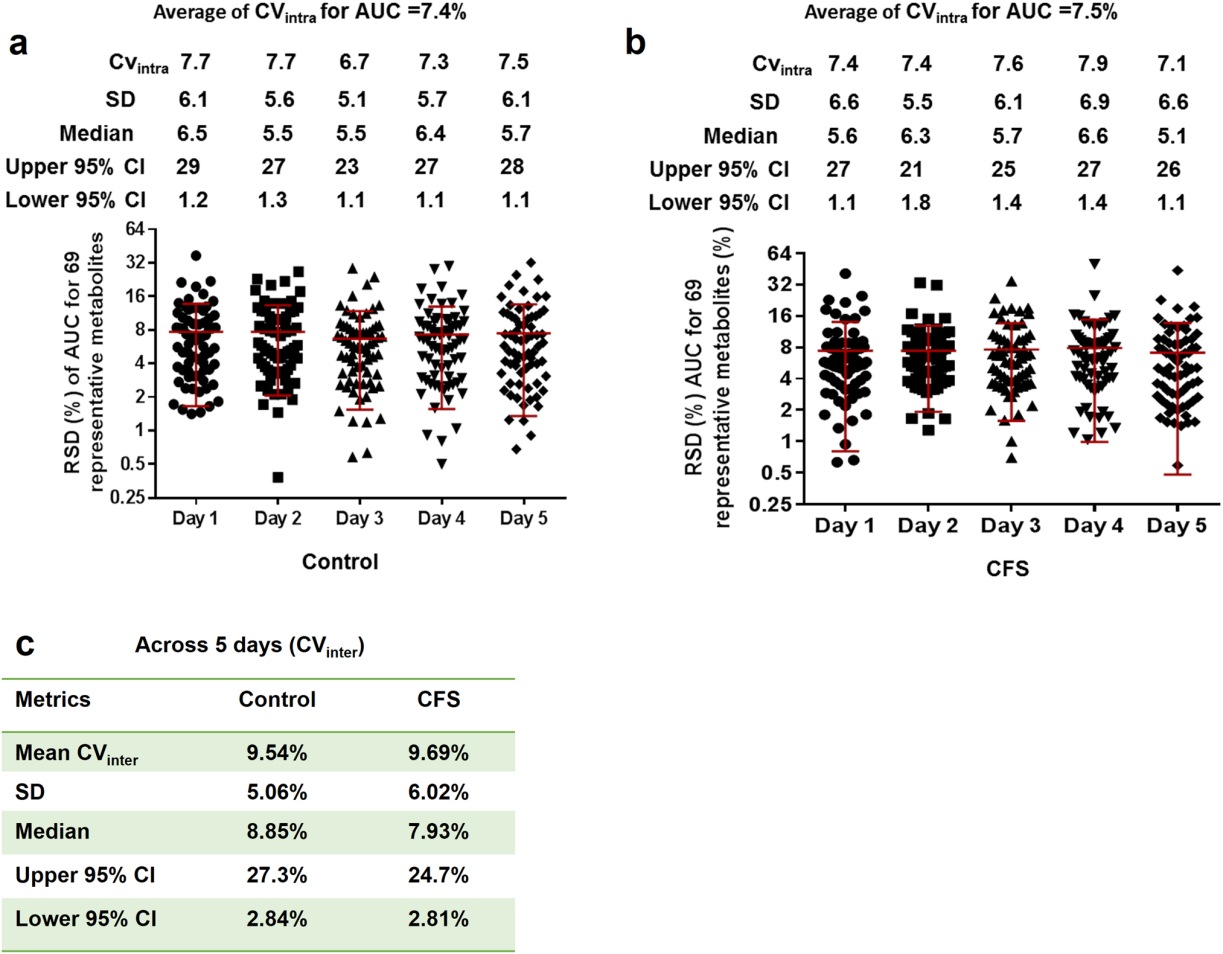
The peak area reproducibility of the developed targeted metabolomic method. **a** The intra-batch CV of the control pool for each of 5 days. **b** The intra-batch CV of the CFS pool for each of 5 days. **c** The inter-batch CV of the control and CFS pool for 5 days. A balanced precision experiment was performed using five replicates of the control and CFS plasma pool analyzed on each of 5 days. The intra and inter-batch CVs for each of the pool were calculated based on 69 representative metabolites. The SD and 95% CI of the CV were also reported

The peak area (AUCs) reproducibility was also excellent. The average CV_intra_ and CV_inter_ of peak area for the control pool was 7.4 and 9.5%, respectively, which was below the acceptance limit (20% RSD) of FDA’s guideline for bio-analytical method validation using LC-MS (Tiwari and Tiwari 2010) (Fig. 4a, c). Similarly, the average CV_intra_ and CV_inter_ of the peak area for the CFS pool was only 7.5 and 9.7% (Fig. 4b, c). The total variability was 12% for both control and the CFS pools.

#### Linearity

**Fig. 5:**
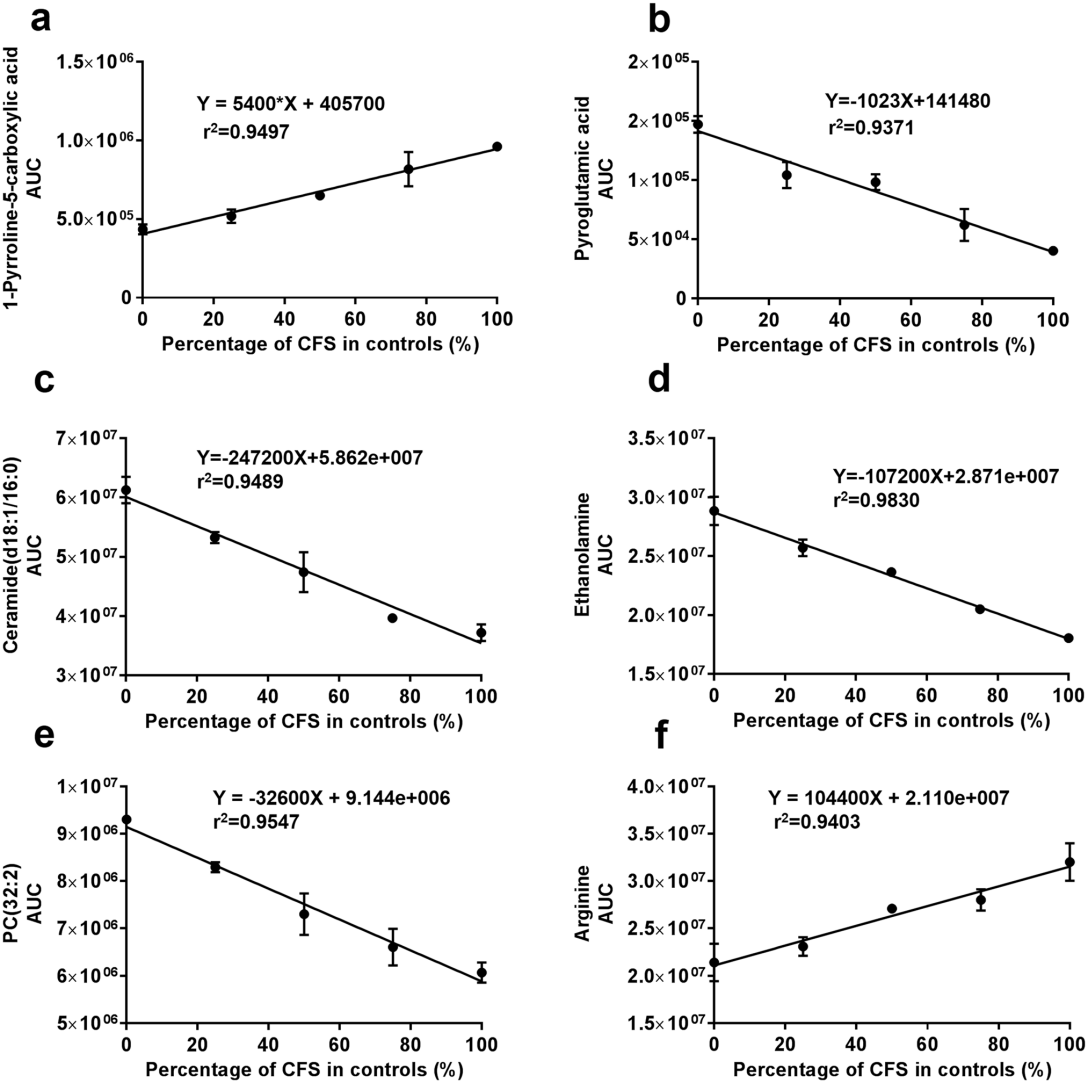
Linearity evaluation. **a**–**f** Six representative metabolites selected for distinguishing male CFS patients from the controls. The linearity was evaluated by mixing the control pool with CFS pool at the ratios of 0:1, 1:3, 1:1, 3:1, and 1:0 ratios. Duplicates were prepared for each point

The linearity was evaluated by mixing the plasma of the healthy controls with CFS patients at 0:1, 1:3, 1:1, 3:1, and 1:0 ratios. Our method showed good linearity with regression coefficients >0.9 for the representative metabolites used for the diagnosis of CFS (Fig. 5a–f). This also indicated that the metabolic biomarkers identified using the developed metabolomic method have the capability for resolving meaningful differences between CFS patients and controls of as little as 20%. Eighty-nine percent (89%) of the detected metabolites had r value >0.90 (Fig. S5).

#### Effect of blood collection tubes

We investigated the differences in metabolomic profile between four different types of blood collection tubes including K_2_-EDTA, Na-heparin, Li-heparin and SSTs. Our results indicated that the anticoagulant has an impact on the detection of plasma metabolites (Fig.S6a). The most striking differences were the significant increase of phosphorylated nucleotides and a 10–50-fold decrease of nucleosides like adenosine in EDTA plasma compared with Li-heparin plasma (Fig.S6c). In contrast, the metabolic differences between Na-heparin and Li-heparin plasma were relatively small (Fig.S6d). In addition, there were dramatic metabolomic differences between serum and plasma with higher levels of eicosanoids, nucleotides and serotonin in serum samples (Fig. S6b and S6d).

#### Carryover

The average carryover, expressed as the AUC percentage of the first blank observed after injection of a sample containing the upper limit of quantification for each analyte, was only 0.75%. Eighty-five percent of the analytes measured had carryovers of less than 2%, and none had more than 4% (Fig. S7).

### Inter-instrument and inter-tester reproducibility

**Fig. 6:**
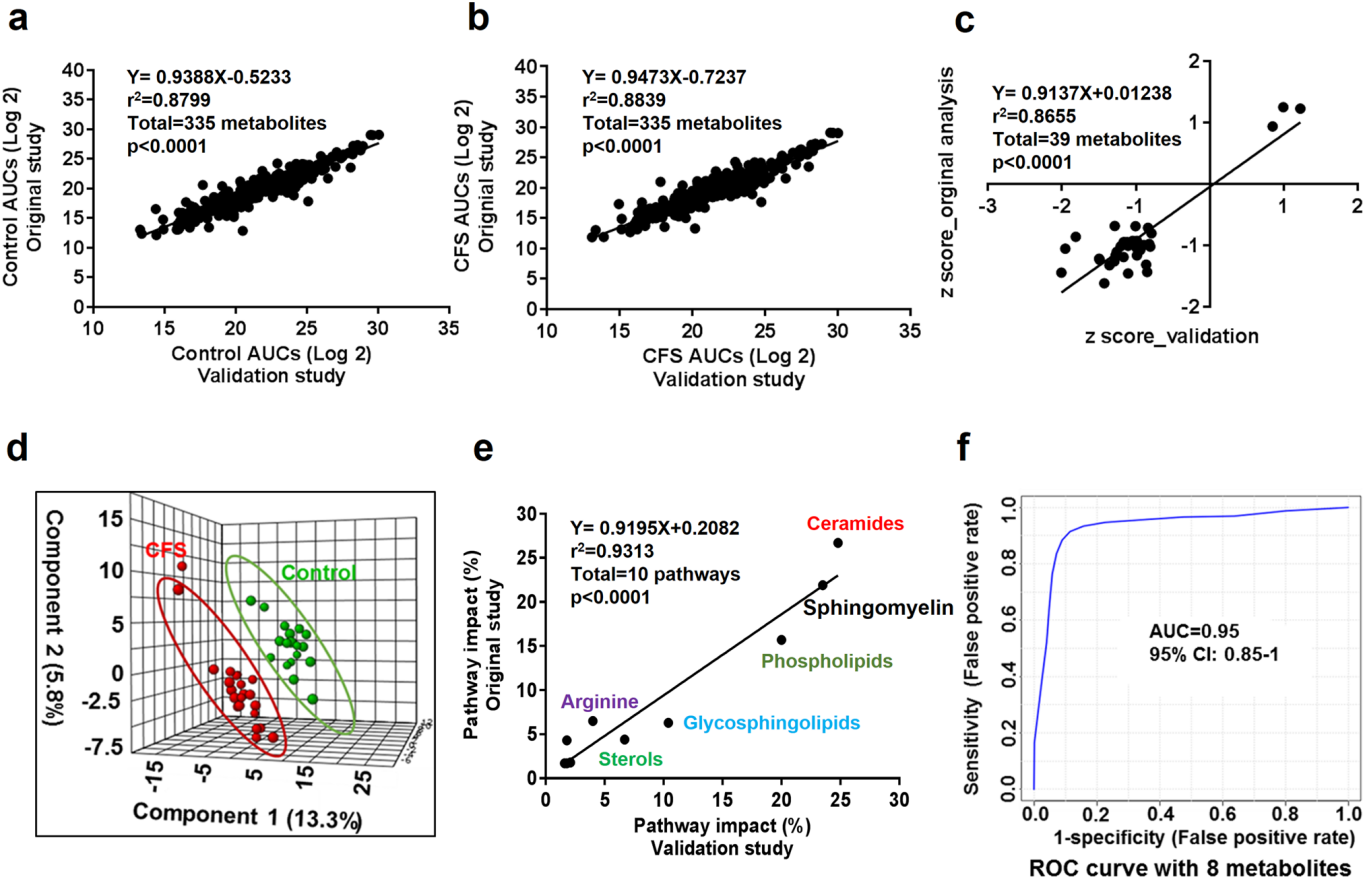
Inter-instrument and inter-tester validation of the developed targeted metabolomic method for its utility in the diagnosis of CFS. **a** AUCs of the control groups in the validation study were correlated with those in the original study (r^2^ = 0.879, p < 0.0001). The AUCs of the targeted metabolites in the plasma extract were obtained in Multiquant 3.0. Data was log 2 transformed prior to Pearson correlation analysis. A total of 335 metabolites were plotted. **b** AUCs of the CFS groups in the validation study were correlated with those in the original study (r^2^ = 0.884, p < 0.0001). **c** The correlation of z-scores between the validation study and the original study (r^2^ = 0.866, p < 0.0001). Metabolites with absolute z-score >0.8 were selected for Pearson correlation analysis. Thirty-nine metabolites were plotted for analysis. **d** PLS-DA analysis showed the clear metabolic differences in plasma between CFS patients and the controls in the validation study. n = 21 male CFS patients and n = 18 matched controls. **e** High correlation of biochemical pathway impact score was achieved between the validation analysis and the original study. 10 out of 14 disturbed metabolic pathways were reproduced in the validation study which account for 94% of the metabolic abnormalities in CFS patients. **f** The diagnostic performance of eight identified biomarkers in the validation study revealed by ROC analysis. The diagnostic accuracy measured as the AUROC curve was 0.95 [95% confidence interval (CI), 0.845–1.0]. The eight metabolites selected were phosphatidyl choline PC (16:0/16:0), glucosylceramide GC (18:1/16:0), 1-pyrroline-5-carboxylic acid (1-P5C), FAD, pyroglutamic acid, Hdroxyisocaproic acid, l-serine, and lathosterol. n = 21 male CFS patients and n = 18 matched controls

To test the inter-instrument and inter-tester reproducibility of our developed metabolomic method which is critical for analytical method transfer, we reanalyzed the 40 CFS and control plasma samples used in our previous study on a different instrument and by a different investigator. The AUCs of 335 metabolites in both control and CFS groups were well correlated between original study and the validation analysis (Fig. 6a, b). Z-score (absolute value >0.8) in the validation study was also well correlated with the previous values (r^2^ = 0.866, p < 0.0001, Fig. 6c). Similar to the original study, PLS-DA in the repeated analysis showed a distinct separation of male CFS from the control group (Fig. 6d). Pathway analysis revealed the significant correlation of pathway impact scores of disturbed pathways in CFS identified by both analyses (Fig. 6e). The 8 metabolite biomarkers identified in our previous study still performed well in distinguishing controls from CFS patients in the validation by AUROC analysis of 95% (95% CI 85–100%) (Fig. 6f).

## Discussion

In this study, we developed, optimized, analytically and clinically validated a robust, broad-spectrum targeted metabolomic workflow based on HILIC-MS/MS (Fig.S8). The key features of our method can be summarized as follows:

### Expanded coverage of the metabolome

Technical improvements in new QqQ instruments include faster scan speeds (as low as 2 ms dwell time for a single transition), rapid polarity switching (≤50 ms), high sensitivity, quantifiability, and large dynamic ranges of 5–6 orders of magnitude, making them ideal for targeted metabolomics. Previously published targeted metabolomic methods typically measure about 200–300 polar metabolites, or require several injections to accommodate positively and negatively charged analytes (Xiao et al. 2012; Zhou et al. 2016). In the present study, we dramatically expanded the metabolome coverage of targeted metabolomics. Using a single injection method, a total of 610 endogenous metabolites and 95 isotopes were targeted. These metabolites covered 63 major metabolic pathways involved in various critical physiological and pathological processes such as glycolysis, TCA cycle, purine and pyrimidine nucleosides and nucleotides, amino acid, fatty acid, phospholipid, and sphingolipid metabolism.

### Powerful separation of diverse classes of metabolites

The greatest challenge in targeted metabolomics comes from the diversity of metabolites that differ greatly in their physical and chemical properties of size, hydrophobicity and charge (Dunn and Ellis 2005). In order to analyze a broader range of metabolites simultaneously, we have focused our efforts on the optimization of LC column separation performance. The separation of cell membrane lipids from the complex biological extract on a HILIC column can be challenging. Glycerophospholipids had been reported to be separated on HILIC columns (Cifkova et al. 2016; Zhu et al. 2012). Our method showed the separation of six different classes of membrane lipids (ceramides, glycosphingolipids, sphingomyelins, cardiolipins, glycerophospholipids bis(monoacylglycero)phosphates), and four subclasses of glycerophospholipids (PC, PI, PE and PS) on the Shodex column in HILIC mode.

The possibility for decreased sensitivity is always a concern when targeting a large number of metabolites in both negative and positive modes simultaneously. The good separation of diverse classes of molecules in our method enabled the quantification of all 705 metabolites in a single run without sacrificing sensitivity. Mean signal to noise ratio was 107, which indicated that the targeted metabolites could be accurately identified.

Our method allowed the baseline separation of some isomers of interest, which previously required chemical derivatization or special columns for accurate quantification. These included alanine and its isomer, sarcosine (Chung et al. 2015), and succinic acid and its isomer, methylmalonic acid (Carvalho and Kok 2008) (Fig. 2e, f). For isomeric species that could not be separated in our method, we measured the total of all molecular species such as the hexose monosaccharide pool (glucose, fructose, galactose, etc).

### Enhanced reproducibility and stability

One of the common challenges with HILIC technique is chromatographic reproducibility (Cubbon et al. 2010). Retained salts from repeated injections of biological samples and the high pH in the mobile phase can potentially cause column degradation in a short period and result in peak broadening and sensitivity reduction (Contrepois et al. 2015). In this study, the polymer-based aminopropyl column from Shodex exhibited excellent intra-batch and long-term inter-batch reproducibility. In addition, the Shodex column was stable under the high pH condition for over 800–1000 injections without significant changes in peak shape, column pressure or sensitivity.

Another factor affecting data reproducibility is the number of points collected across the peak. Scanning too many metabolites at the same time can increase the cycle time and reduce the points across the peak, which in turn reduces peak area reproducibility. In our method, the gradient was carefully optimized so that even in the most intense scanning period in the gradient (RT 16–18 min), only about 150 metabolites were simultaneously targeted by the scheduled MRM algorithm. The average number of points across the peak were 20, which were enough for the generation of reproducible peak area each time.

### Selection of sample collection tubes

Bias in sample collection is a frequently forgotten problem responsible for uncontrolled errors in metabolomic analysis and can have a major impact on the identification of biomarkers. Our results showed clear differences between K_2_-EDTA and Li-heparin plasma that related to the specific class of metabolites measured. The choice of tubes should therefore be made based on the aim of the study. If the study focuses on the analysis of phosphorylated nucleotides, EDTA tubes have some advantages, but come at the cost of several weaknesses. For more general, targeted, metabolomic analysis across many different classes of molecules, Li-heparin tubes are recommended. Our findings are in line with others who have recommended EDTA plasma for untargeted studies (Yin et al. 2013) and Li-heparin plasma for targeted studies (Dunn et al. 2011). Serum samples, which avoid the issue of chemical additives, but require a clotting step, may also have utility in some metabolomics studies. The coagulation cascade during the preparation of serum affected the levels of certain classes of metabolites such as eicosanoids, nucleotides and serotonin. When using archived samples from biobanks, critical attention should be paid to the type of collection tube that was used. Collection tube chemistry must be standardized for each metabolomic experimental series.

### Inter-instrument and inter-tester validation for disease diagnosis

This study successfully replicated our recent findings of the unique chemical signature in CFS patients, which could be used for the diagnosis of CFS (Naviaux et al. 2016). Although the samples had been stored in −80 °C for about 1.5 years and a small number of metabolites might be changed because of degradation or ion suppression effects, the chemical signature and the diagnostic biomarkers remained unchanged. Good linearity was still achieved for these biomarkers in the 5-point mixing experiment. To validate the method independently, we repeated the analysis on this 40-sample study on a different Qtrap 5500 system by a different investigator. Even though nearly 95% of the absolute peak areas were different due to sensitivity and tuning differences between two instruments, the eight metabolites selected in our original study still performed with a 95% ROC curve accuracy in distinguishing controls from CFS patients (Fig. 6). And perhaps even more importantly, pathway analysis of the results validated the original study results, emphasizing the importance of sphingolipids and phospholipids in the biology of CFS in this cohort (Naviaux et al. 2016).

### Analytical limitations

A mass resolution of ±0.7 da is a known limitation of QqQ mass spectrometers, which rely on characteristic fragmentation patterns to identify metabolites. Occasionally, isobaric molecules cannot be resolved in our method such as hexose sugars. The hexose pool was quantified. Peak splitting was observed for some of long-chain acylcarnitines such palmitoylcarnitine (C16) and myristoylcarnitine (C14) on the HILIC column. In addition, some neurotransmitters (norepinephrine, epinephrine and melatonin) were not well detected in our method. A reverse phase method might be an alternative solution for some of these metabolites.

## Conclusion

We report an optimized method for targeted, broad-spectrum metabolomic analysis of 610 molecules from over 60 biochemical pathways in a single injection. This method performed well in rigorous analytical validation and was able to reproduce the metabolomic results from samples stored for over 1.5 years in a clinical validation experiment.

## Electronic supplementary material

Below is the link to the electronic supplementary material.

Supplementary material 1 (DOCX 2339 KB)

Supplementary material 2 (XLSX 141 KB)
