# Supplementary material for: A robust, single-injection method for targeted, broad-spectrum plasma metabolomics
Source: Metabolomics. 2017 Sep 4;13(10):122. doi: 10.1007/s11306-017-1264-1 (PMC5583274; doi:10.1007/s11306-017-1264-1)
Supplement: Supplementary file 1 — Supplementary material 1 (DOCX 2339 KB) [file 11306_2017_1264_MOESM1_ESM.docx]

**ONLINE SUPPLEMENTAL MATERIALS**

**A Robust, Single-injection Method for Targeted,**

**Broad-spectrum Plasma Metabolomics**

Kefeng Li^1,2*^, Jane C. Naviaux^1,5^, A. Taylor Bright^1,2^, Lin Wang^1,2^, Robert K. Naviaux^1-4*^

^1^The Mitochondrial and Metabolic Disease Center, University of California, San Diego, School of Medicine, San Diego, CA, 92103-8467, USA

^2^Department of Medicine, University of California, San Diego, School of Medicine, San Diego, CA, 92103-8467, USA

^3^Department of Pediatrics, University of California, San Diego, School of Medicine, San Diego, CA, 92103-8467, USA

^4^Department of Pathology, University of California, San Diego, School of Medicine, San Diego, CA, 92103-8467, USA

^5^Department of Neurosciences, University of California, San Diego, School of Medicine, San Diego, CA, 92103-8467, USA

*Communicating authors: [kli@ucsd.edu](mailto:kli@ucsd.edu), and [naviaux@ucsd.edu](mailto:naviaux@ucsd.edu)

**Acknowledgments:** The authors thank Jonathan Monk for computational and bioinformatics assistance. This work was supported by gifts from the UCSD Christini Fund, the Lennox Foundation, The Wright Family Foundation, the UCSD Mitochondrial Research Fund, and the Jane Botsford Johnson Foundation.

**Contents**

[**1. Supplemental Materials and Methods** 3](#_Toc491437325)

[**1.1 Quality control (QC) samples** 3](#_Toc491437326)

[**1.2 Metabolites extraction** 3](#_Toc491437327)

[**1.3 Procedures for optimization of LC conditions** 3](#_Toc491437328)

[**1.3.1 Column selection** 3](#_Toc491437329)

[**1.3.2 Mobile phase composition and pH** 3](#_Toc491437330)

[**1.3.3 Oven temperature** 4](#_Toc491437331)

[**1.4 Procedures for the optimization of MS/MS conditions** 4](#_Toc491437332)

[**1.4.1 Electrospray source temperature** 4](#_Toc491437333)

[**1.4.2 Ion source optimization** 4](#_Toc491437334)

[**1.4.3 Influence of rapid polarity switch** 5](#_Toc491437335)

[**1.5 Optimization of compound-dependent MRM parameters** 5](#_Toc491437336)

[**1.6 Procedures for analytical validation** 5](#_Toc491437337)

[**1.6.1 Reproducibility evaluation** 5](#_Toc491437338)

[**1.6.2 Linearity evaluation** 5](#_Toc491437339)

[**1.6.3 Peak quality assessment** 5](#_Toc491437340)

[**1.6.4 Effect of blood collection tubes** 6](#_Toc491437341)

[**1.6.5 Carryover assessment** 6](#_Toc491437342)

[**2. Supplemental Results** 6](#_Toc491437343)

[**2.1 Optimization of mobile phase, pH conditions and oven temperatures** 6](#_Toc491437344)

[**2.2 Optimization of electrospray (ESI) source temperature and gas** 7](#_Toc491437345)

[**3. Supplemental Tables** 8](#_Toc491437346)

[**4. Supplemental Figures** 9](#_Toc491437347)

# **1. Supplemental Materials and Methods**

## **1.1 Quality control (QC) samples**

Adult human plasma was pooled together. Ninety (90) µl of plasma was mixed with 5 µl of commercial internal standards and 5 µl of custom-synthesized ^13^C labeled standards and then incubated for 10 min at room temperature. The metabolites were then extracted with 400 µl of cold (-20°C) MEOH: ACN (50:50). The samples were then vortexed vigorously, incubated on crushed ice for 10 min, and then centrifuged at 16,000 g for 10 min at 4 °C. The supernatants were transferred to labeled cryotubes and stored at -80 °C as quality control samples (QC). The QC samples were used for method optimization, analytical validation and batch quality control.

## **1.2 Metabolites extraction**

Plasma samples (90 µl) were mixed with 5 µl of commercial internal standards and 5 µl of custom-synthesized ^13^C labeled standards and then incubated for 10 min at room temperature to permit small molecules and vitamins in the internal standards to associate with plasma binding proteins. The metabolites were extracted with 400 µl of cold (-20°C) methanol: acetonitrile (50:50 MEOH: ACN), yielding a final concentration of 40/40/20% methanol: acetonitrile: water. The samples were then vortexed vigorously, incubated on crushed ice for 10 min, and then centrifuged at 16,000 g for 10 min at 4 °C. The supernatants containing the extracted metabolites and internal standards were transferred to labeled cryotubes and stored at -80 °C prior to LC-MS/MS.

## **1.3 Procedures for optimization of LC conditions**

### **1.3.1 Column selection**

Aminopropyl columns from three different manufacturers: Luna NH2 (Phenomenex), inertsil NH2 (GL Science) and Asahipak NH2P-40 (Shodex) were evaluated. Columns of different dimensions were also tested including 50×2, 150×2, and 250×2mm.

### **1.3.2 Mobile phase composition and pH**

Six different mobile phase compositions and pH conditions were tested: (1) 20 mM NH_4_COOH+0.1% formic acid, pH 3.6; (2) 20 mM NH_4_OAC, pH 6.8; (3) 20 mM NH_4_OH+20 mM FA, pH 9.4; (4) 80 mM NH_4_OH+20 mM formic acid, pH 10; (5) 15 mM NH_4_HCO_3_+100 mM NH_4_OH, pH 10; (6) 20 mM (NH_4_)_2_CO_3_+31mM NH_4_OH, pH9.8. Retention time, AUC and peak width were obtained with Multiquant 3.0. The score of each metabolite was calculated using the following score system:

| **AUC** | **Score_AUC_** | **Width** | **Score**_width_ |
| --- | --- | --- | --- |
| AUC<1×10^4^ | -3 | Width<0.5 min | 1 |
| 1×10^4^ <AUC<5×10^4^ | -1 | 0.5<Width<1 min | 0 |
| 5×10^4^ <AUC<2×10^5^ | 0 | 1<Width<2.5 min | -1 |
| AUC>2×10^5^ | 1 | Width>2.5 min | -3 |

| Score_total_ | >0 | Good |
| --- | --- | --- |
|  | 0 | Acceptable |
|  | <0 | Bad |

Score_total_=Score_AUC_ + Score_width_.

### **1.3.3 Oven temperature**

Oven temperature was optimized and five temperatures including 20, 25, 30, 35 and 40 ºC were tested using the QC samples. Retention time, AUC and peak width were obtained with Multiquant 3.0. The score of each metabolite was calculated using the above score system.

## **1.4 Procedures for the optimization of MS/MS conditions**

### **1.4.1 Electrospray source temperature**

The effect of source temperature on the detection of diverse classes of targeted metabolites was evaluated. Three temperatures including 400 ºC, 500 ºC and 600 ºC were tested using the QC samples. Five replicates were set up for each temperature condition. The peak integration and AUCs were generated in Multiquant 3.0 (SCIEX). Heatmap correlation analysis was conducted in metabonalyst (www. metaboanalyst.ca) and the top 60 metabolites calculated from ANOVA were listed.

### **1.4.2 Ion source optimization**

Ion source gas of 25 psi (172.4 kpa of 99.5% of nitrogen), 30 psi (206.8 kpa), 35 psi (241.3 kpa) and 40 psi (275.8 kpa) were tested. Five replicates were set up for each gas setting. The peak integration and AUCs were generated in Multiquant 3.0 (SCIEX). Heatmap correlation analysis was conducted in metabonalyst (www.metaboanalyst.ca) and the top 60 metabolites calculated from ANOVA were listed.

### **1.4.3 Influence of rapid polarity switch**

The effect of polarity switch on the overall detection of the targeted metabolites was investigated. Briefly, the QC plasma extract was analyzed using either polarity switch or single polarity. The polarity switch time was set at 50 ms in analyst 1.6 software (SCIEX). For single polarity, two injections were conducted: one for negative mode and the other one for positive mode. Total 707 metabolites were targeted and 468 were detected in QC plasma. The peaks were integrated and AUCs were generated in Multiquant 3.0 (SCIEX). The AUCs were log2 transformed and the correlation between two approaches were calculated using Pearson analysis and linear regression analysis in Prism 6.0 (Graphpad Prism).

## **1.5 Optimization of compound-dependent MRM parameters**

The compound-specific MRM transitions were optimized using the authentic standards. To ensure the analytical specificity and accuracy, we optimized the MRM transitions and verified the retention time for each metabolite using two different concentrations (1x and 10x) of purified standards in the pure solvent and pooled plasma matrix. Table S1 listed the optimized MRM transitions, compound-specific MS/MS parameters, retention time and other related information.

## **1.6 Procedures for analytical validation**

### **1.6.1 Reproducibility evaluation**

A balanced precision experiment was performed to evaluate the reproducibility of the method. The controls and CFS samples were pooled together. Five replicates were analyzed for each of the control and CFS pool for 5 days. The mean intra-assay (CVintra), inter assay (CVinter) and total CVs for each pool were calculated.

### **1.6.2 Linearity evaluation**

The linearity was determined using 5-point admixtures of control and CFS pools. 100%, 75%, 50%, 25%, and 0% of the CFS pool were mixed with the control pool. The mixture was analyzed using the developed method. Duplicates injections were performed for each point.

### **1.6.3 Peak quality assessment**

The peak quality metrics for each detected metabolite were generated in Multiquant 3.0 including peak width, peak width at 50% height, points across the peak and signal to noise ratio (S/N). Histograms and nonlinear fit of the histograms (Gaussian distribution) were plotted in Prism 6.0 (Graphpad Prism) in log space and the mean and 95% confidence interval (CI) were provided in the linear space.

### **1.6.4 Effect of blood collection tubes**

Blood was collected from 5 volunteers into four different types of tubes including lithium heparin (Li-hep), sodium heparin (Na-hep), K2-EDTA and golden serum separator tubes (SSTs). Plasma was separated by centrifugation at 900 g x 10 min at room temperature within one hour of collection. Blood in SSTs was incubated at room temperature for 30 min and the serum was obtained by centrifugation at 900 g for 10 min at room temperature. Metabolites were extracted from the resulting plasma and analyzed in triplicates using the developed targeted metabolomic method.

### **1.6.5 Carryover assessment**

Carryover was assessed by injecting a blank sample after a high concentration sample (Plasma QC spiked with standards to reach the upper limit of quantification (ULOQ) for most analytes). The percentage of metabolites residue was calculated by the ratio of AUC of the analyte in the blank sample to the analyte in the high concentration sample (AUC_blank_/AUC _high concentration analyte_). Total 71 representative metabolites covering the entire gradient were selected for evaluation.

# **2. Supplemental Results**

## **2.1 Optimization of mobile phase, pH conditions and oven temperatures**

The mobile phase compositions, pH and salt concentrations determine the ionic strength of the mobile phase and have dramatic impacts on metabolite separation and MS ionization efficiency. We tested a range of pH conditions (pH 3.6, 6.8, 9.44, 9.8 and 10) and salts (ammonium formate, ammonium acetate, ammonium hydroxide, ammonium bicarbonate and ammonium carbonate) for metabolites separation, peak shape and MS sensitivity using pooled plasma QC samples. Acidic and neutral pH had weak ionic strength on the polymer-based aminopropyl HILIC column, which yielded bad peak shape or no detection for amphiphatic and polar metabolites such as chenodeoxyglycocholic acid, TTP and ATP (Fig. S1). The nucleotide triphosphates were detected only in basic pH mobile phase on the Shodex column, with the addition of either ammonium bicarbonate or ammonium carbonate (Fig. S1). To facilitate the comparison of the different mobile phase conditions, we further selected a set of 86 metabolites with diverse chemical properties and assigned a score for each metabolite based on its sensitivity and peak shape (Fig. S2). The sum of the scores for each chromatographic condition was calculated and divided into three categories: good (Score_total_ >0, green color), acceptable (Score_total_ =0, brown color) and bad (Score_total_ <0, red color). Overall, 20 mM (NH4)_2_CO_3_+31 mM NH_4_OH at pH 9.8 had the highest number of metabolites (93%) with good or acceptable scores and thus was the optimal mobile phase condition (Fig. S2a).

We next evaluated the impact of column temperature on the overall detection and peak shape of the targeted metabolites. A range of oven temperatures from 20 ^◦^C to 40 ^◦^C was assessed (Fig. S2b). Both 25 ^◦^C and 30 ^◦^C had a higher number of good and acceptable peaks than other conditions. We ultimately chose 25 ^◦^C to minimize the potential degradation of stationary phase, which might be accelerated at elevated temperature, an important consideration when also operating at high pH.

## **2.2 Optimization of electrospray (ESI) source temperature and gas**

Generally, optimization of ESI interface settings for the single analyte is carried out by infusion of a standard solution into the ionization source. This is impossible for the optimization of a large set of metabolites in metabolomics since the optimal parameters might be different for different analytes and the potential interactions between metabolites in the matrix can interfere with the optimization. We optimized the source temperature and ion source gas (sheath gas and drying gas) using QC plasma extract to determine the conditions suitable for our diverse classes of targeted metabolites. Optimal source temperature was set at 500 ºC, as shown in Fig. S3a. Of note, a source temperature of 500 ºC allowed good detection of both polar metabolites and lipids, without the dramatic decrease in lipid detection observed at 600 ºC, or the decrease in polar metabolites observed at 400 ºC.

Ion source gas is critical for the formation of electrospray droplets and the detection of the analytes. The overall peak intensities at gas 35 and 40 psi were higher than the gas settings of 25 and 30 psi (Fig. S3b). We then selected 35 psi for ion source gas to avoid the potential fragmentation of thermally unstable metabolites.

# **3. Supplemental Tables**

**Table S1: The detailed information of 707 targeted analytes (MRM transitions, DP, CE, chemical formular, HMDB numbers, chemical classes, biological pathways, LoD and LoQ)**

**Check the excel file.**

**Table S2: High inter-instrument and inter-tester reproducibility of the developed metabolomic method**

| **Classifier** | **Algorithm model** | **AUROC** | **95% CI** | **rdCV accuracy^++^** | **Permutation p value^+++^** | **Sensitivity*** | **Specificity*** |
| --- | --- | --- | --- | --- | --- | --- | --- |
| The same 8 analytes in this study | Random forests | 0.96 | 0.85-1 | 0.92 | 0.001 | 0.95 | 0.89 |
| Eight analytes in our previous study^+^ | Random forests | 0.94 | 0.84-1 | 0.84 | 0.001 | 0.91 | 0.89 |

+: Naviaux, R. K., Naviaux, J. C., Li, K., Bright, A. T., Alaynick, W. A., Wang, L., et al. (2016). Metabolic features of chronic fatigue syndrome. Proc Natl Acad Sci USA, 113(37), E5472-5480, doi:10.1073/pnas.1607571113.

++: rdCV, repeated random subsample (2/3 in, 1/3 out) double cross-validation.

+++: Permutation P values represent the probability that the RF classification of cases and controls using the specified analytes could be obtained by chance.

*Values calculated by standard 2 × 2 contingency table analysis.

# **4. Supplemental Figures**


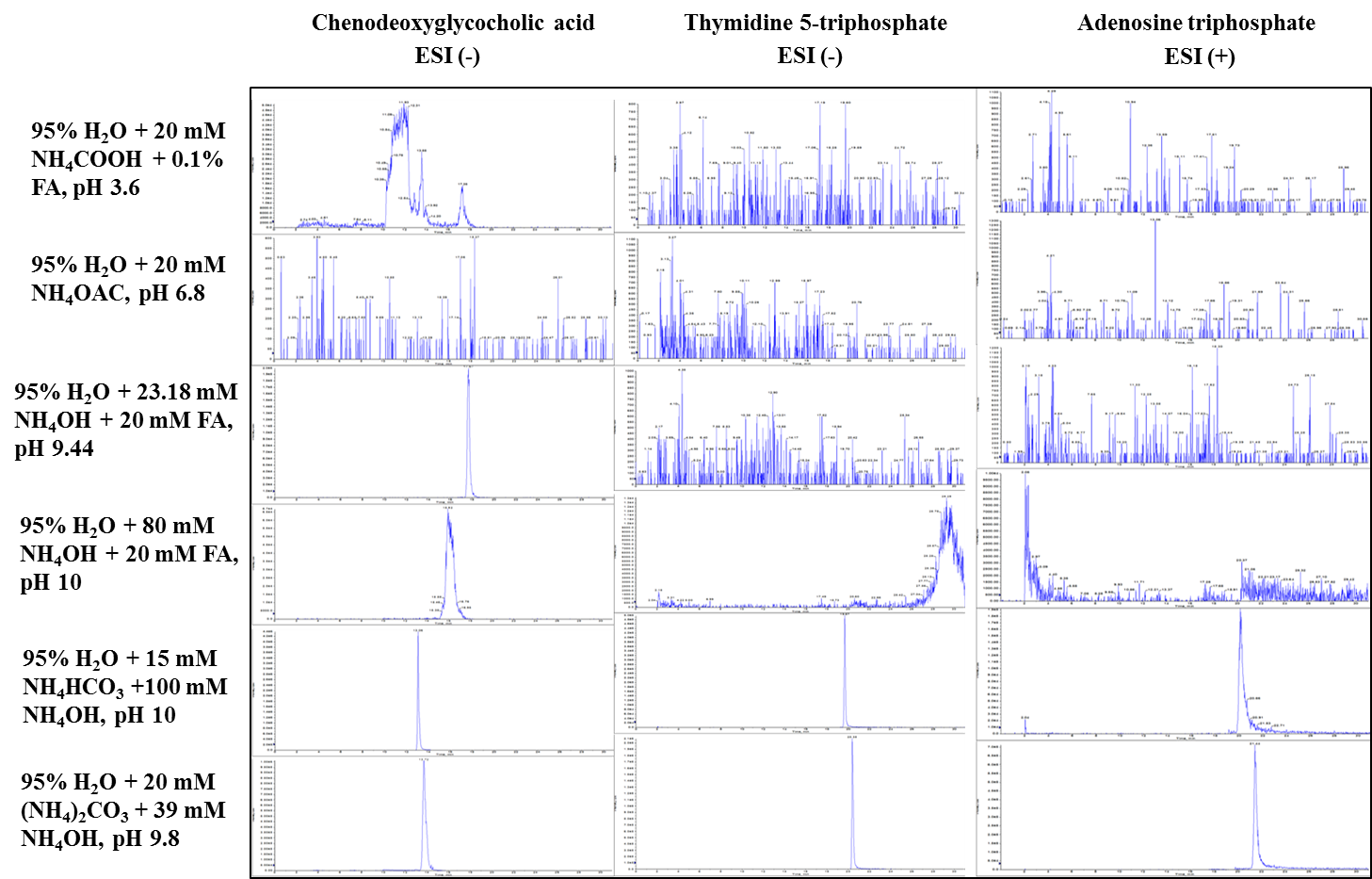


**Fig. S1 Extracted ion chromatograms of selected metabolites under different mobile phases.**


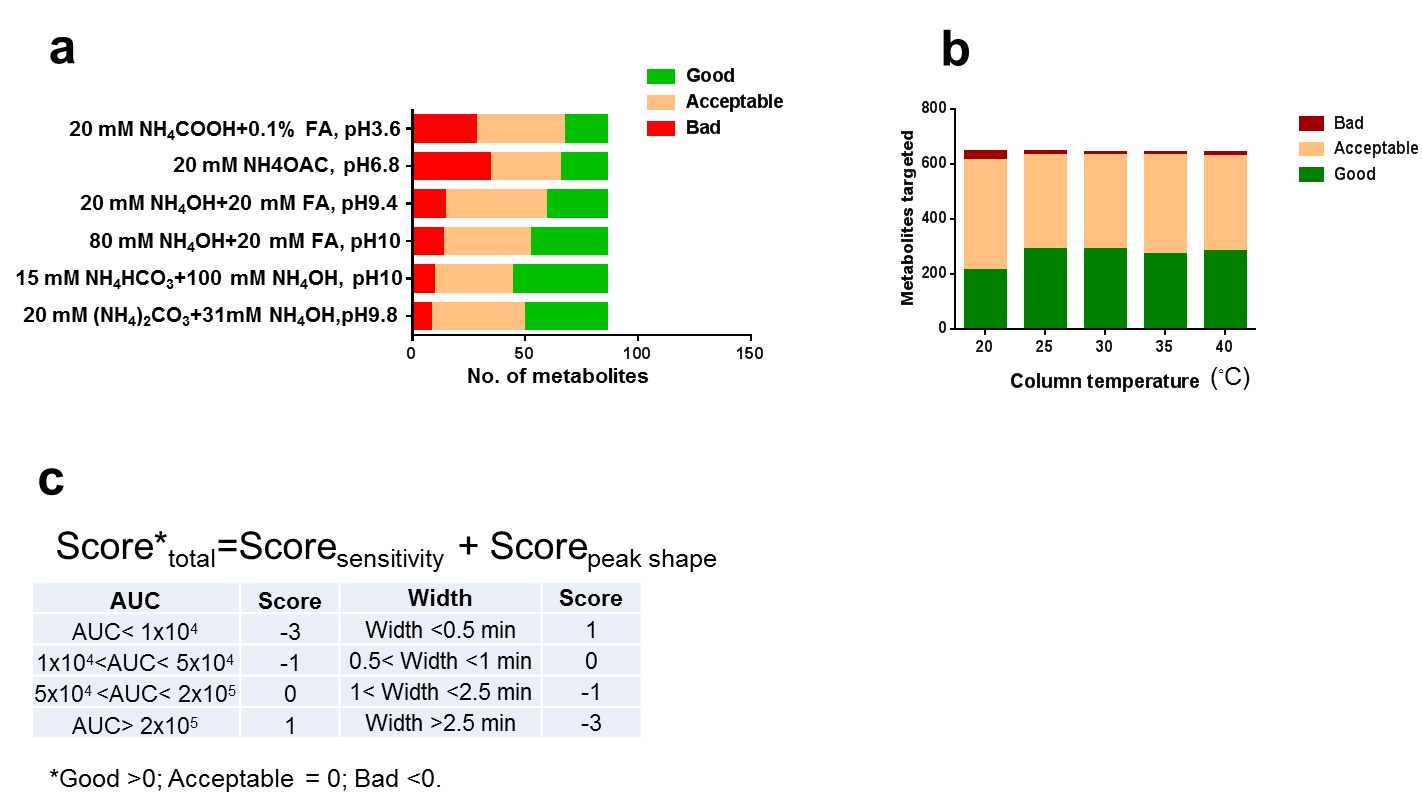


**Fig. S2 Optimization of LC conditions.**

(a): Effect of different mobile phases and pH conditions. (b): Effect of column temperature.


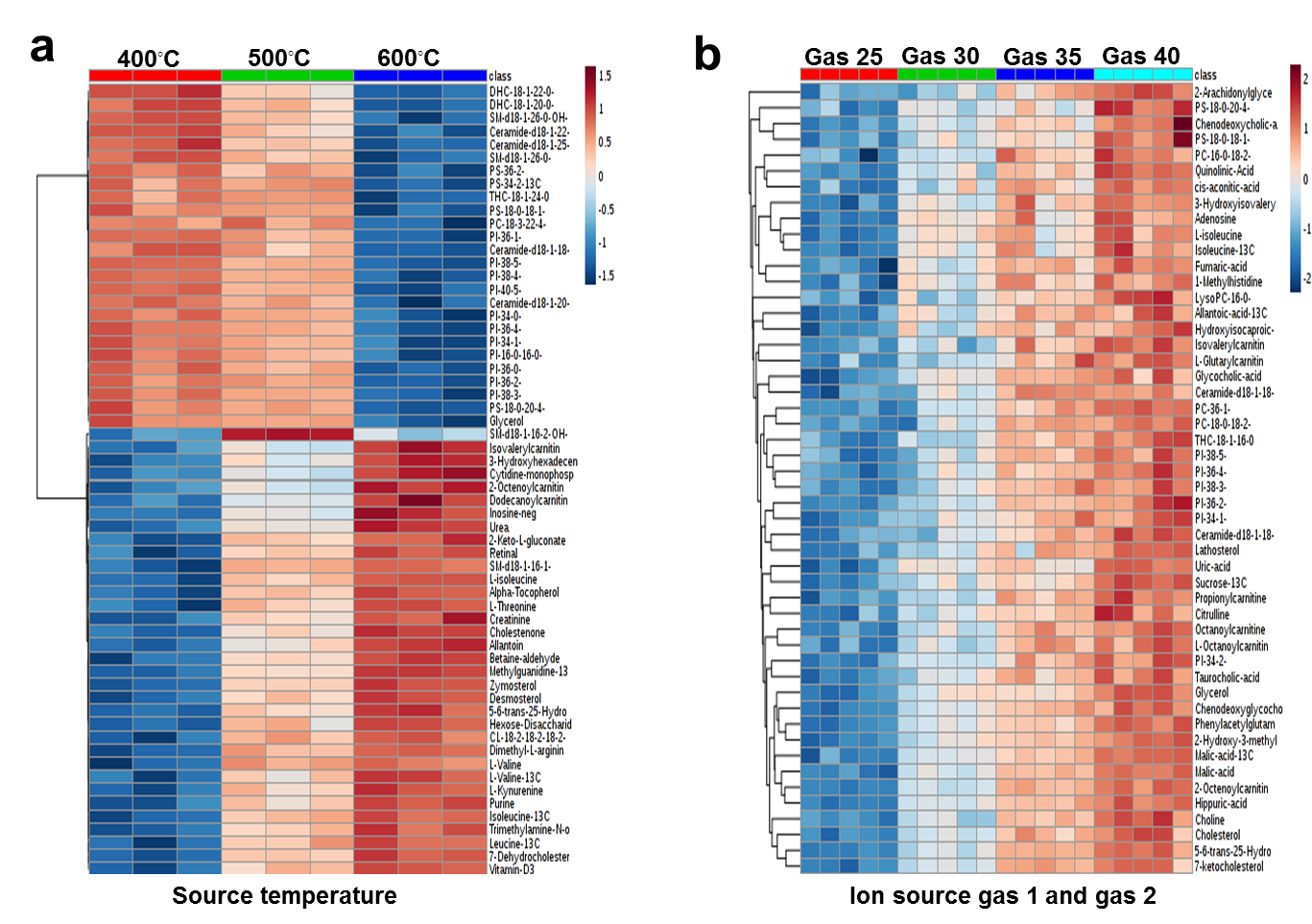


**Fig. S3 Optimization of ESI source parameters.**

(a) Source temperature. 400◦C, 500◦C and 600◦C were tested and 3 replicates were set up for each temperature. (b) Ion source gas (nebulizer gas). 25, 30, 35 and 40 were tested for both gas 1 and gas 2. Five replicates were set up for each ion source condition. The top 60 metabolites calculated from ANOVA were listed. Euclidean classification and ward clustering algorithm were used for correlation analysis.


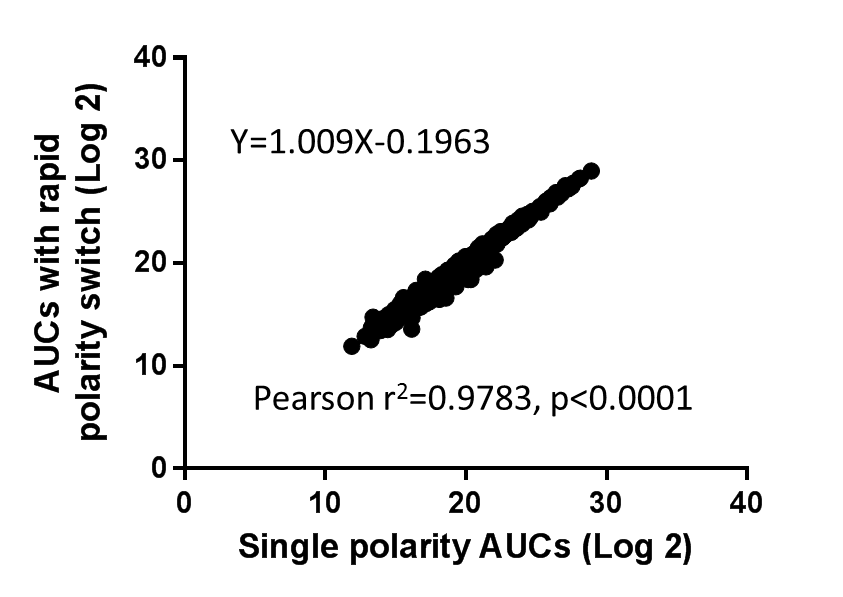


**Fig. S4 Rapid polarity switch improves the high-throughput of metabolomics without dramatically reducing the sensitivity.**

The same QC plasma extract was run using either polarity switch or single polarity (one injection for negative mode and the other one for positive mode) approach. Total 707 metabolites were targeted and 468 were detected in QC plasma. The AUCs were log 2 transformed and the correlation between two approaches were calculated using Pearson analysis and linear regression analysis. The polarity switch time was set at 50 ms for Qtrap 5500 instrument.


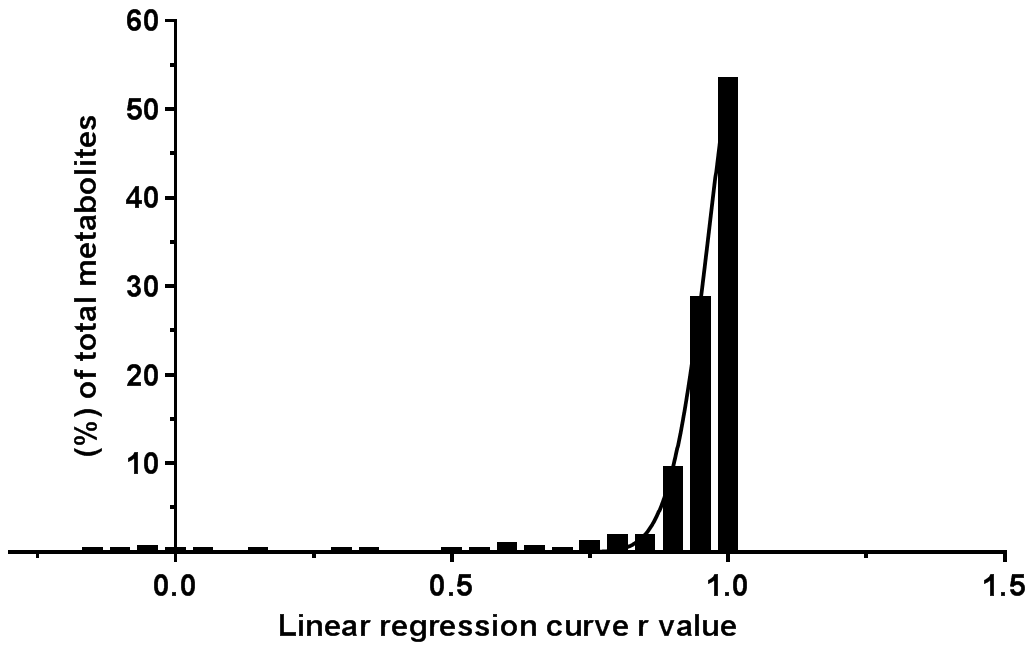


**Fig. S5 The distribution of linearity r for the detected metabolites in pooled human plasma.** The plasma QC samples were diluted for 1:2, 1:4, 1:8; 1:16; 1:32; 1:64 and 1:128 before extraction. Linear regression analysis was conducted. Eight-nine percent (89%) of metabolites detected had r>0.90.


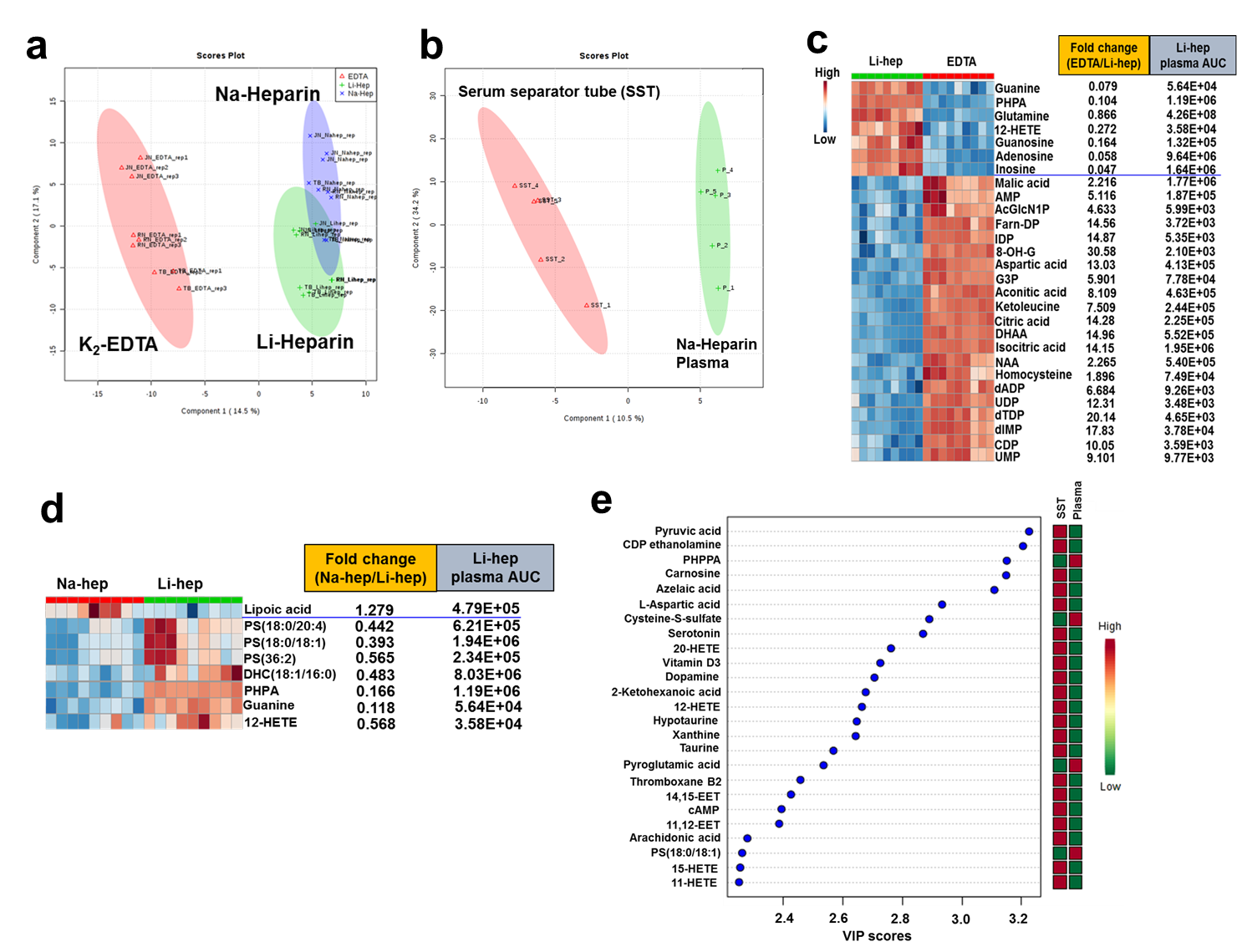


**Fig. S6 Effect of blood collection tubes on the metabolomic analysis**.

(a): PLS-DA showed the complete separation of metabolomic profile of EDTA plasma from heparin plasma. (b): The separation of metabolomic profile between serum and Na-heparin plasma revealed by PLS-DA. (c): The comparison of EDTA tube and Li-hep tube. Out of 450 metabolites detected, 70 metabolites (15.6%) were significantly different between EDTA and Li-hep plasma. The top 28 metabolites ranked by p value were listed. (d): The comparison of Na-hep and Li-hep tubes. The cut-off values were fold change>1.2, p<0.05 and FDR<0.1. Only 8 metabolites (1.8%) were significantly different between Na-hep and Li-hep. (e): The top 25 differential metabolites between serum and Na-heparin plasma. Blood was collected from 5 volunteers in four different types of blood collection tubes including lithium heparin (Li-hep), sodium heparin (Na-hep), EDTA tubes and golden serum separator tube. The plasma and serum was prepared according to the standard protocol. Metabolites were extracted and analyzed using the developed metabolomic method.


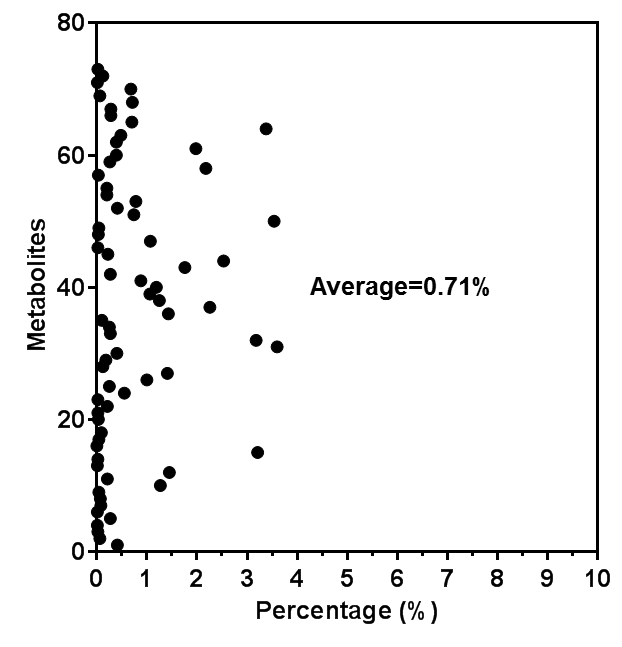


**Fig. S7 Carryover evaluation**.

Carryover was assessed by injecting a blank right after a plasma QC sample spiked with standards to reach the upper limit of quantification (ULOQ) for most analytes. The percentage of metabolites residue was calculated by the ratio of AUC of the analyte in the blank sample to the analyte in the high concentration QC sample (AUC_blank_/AUC _high concentration analyte_). The average carryover was calculated using 63 representative metabolites.


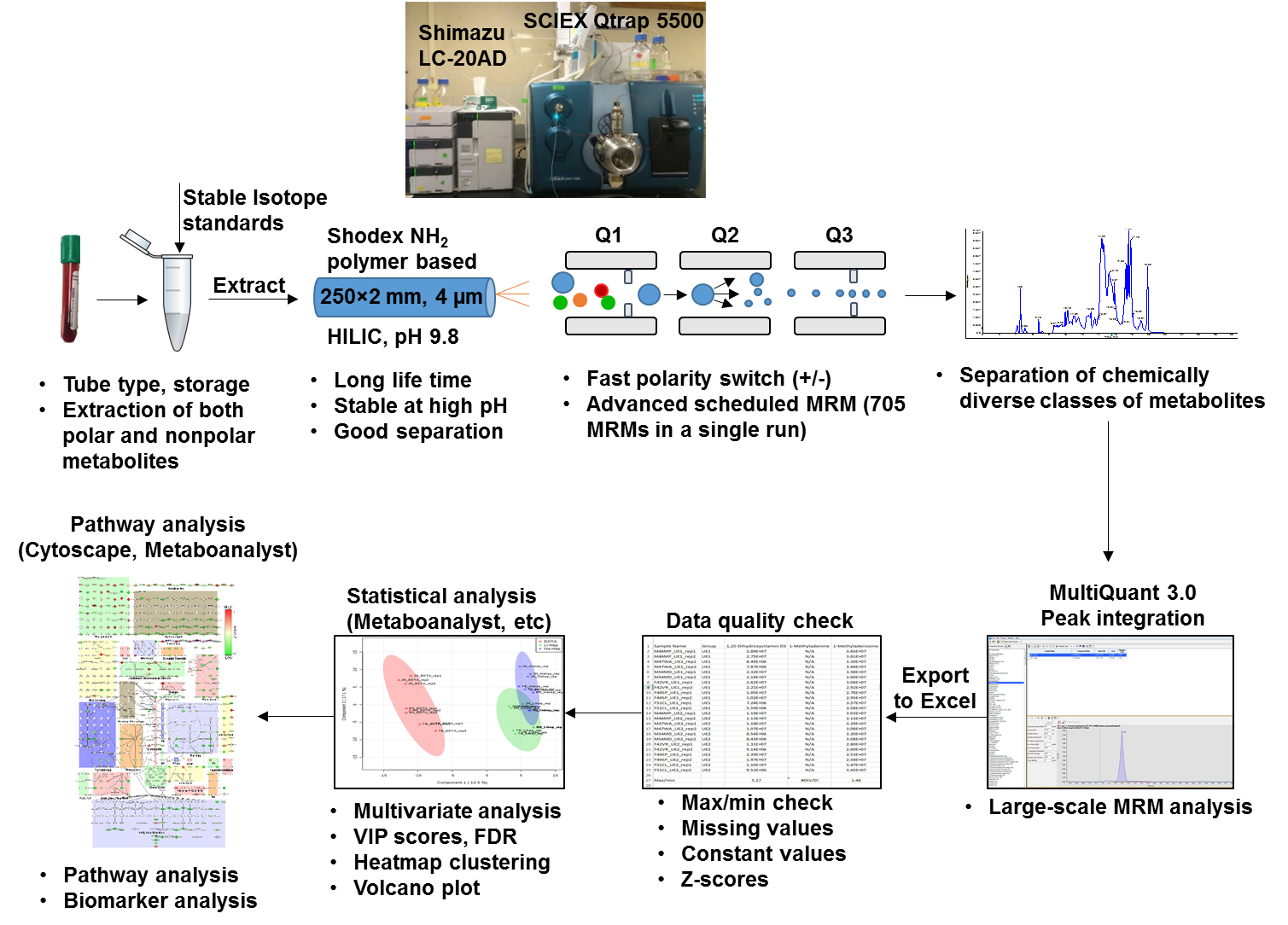


**Fig. S8 Metabolomics Workflow.** Sample collection to pathway and biomarker analysis.
